# Supplementary figures and images for: Sex-, age-, and organ-dependent improvement of bile acid hydrophobicity by ursodeoxycholic acid treatment: A study using a mouse model with human-like bile acid composition
Source: PLoS One. 2022 Jul 12;17(7):e0271308. doi: 10.1371/journal.pone.0271308 (PMC9275687; doi:10.1371/journal.pone.0271308)

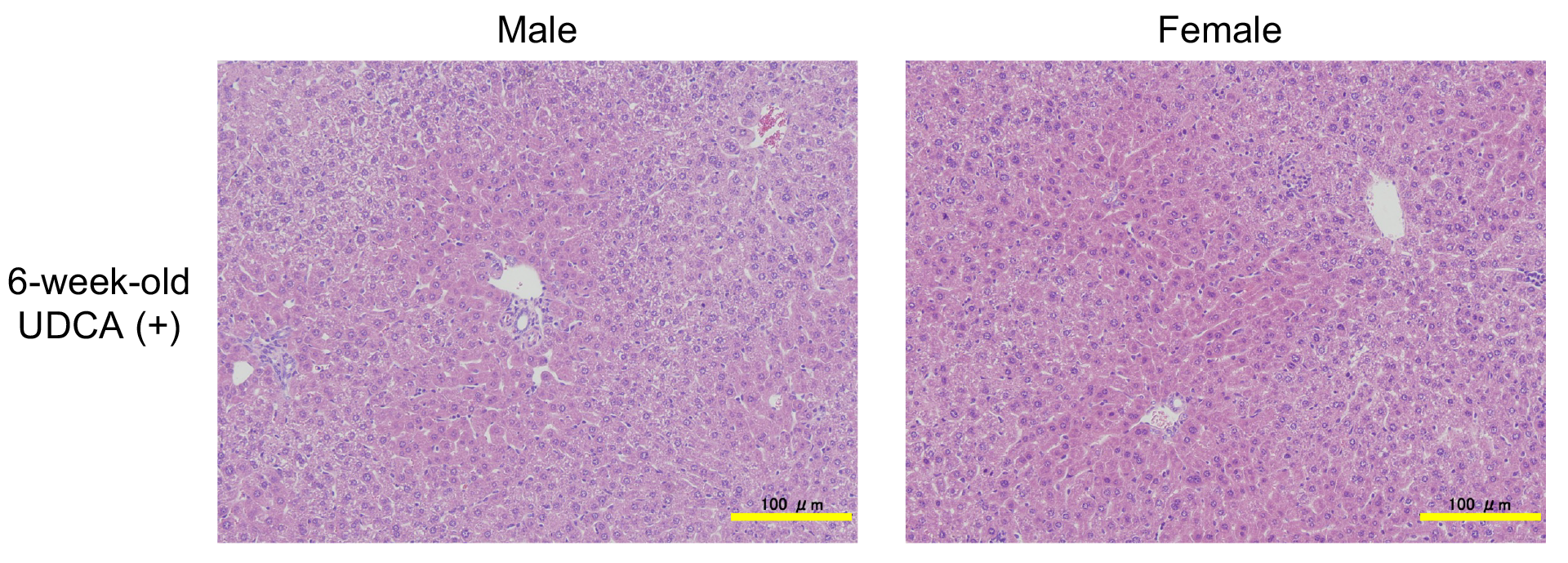

Supplement: S1 Fig — Both male and female UDCA-treated 6-week-old DKO mice showed no significant histological findings. Hematoxylin/eosin stain. Scale bars, 100 μm. UDCA (+), with UDCA. (TIF) [file pone.0271308.s002.tif]

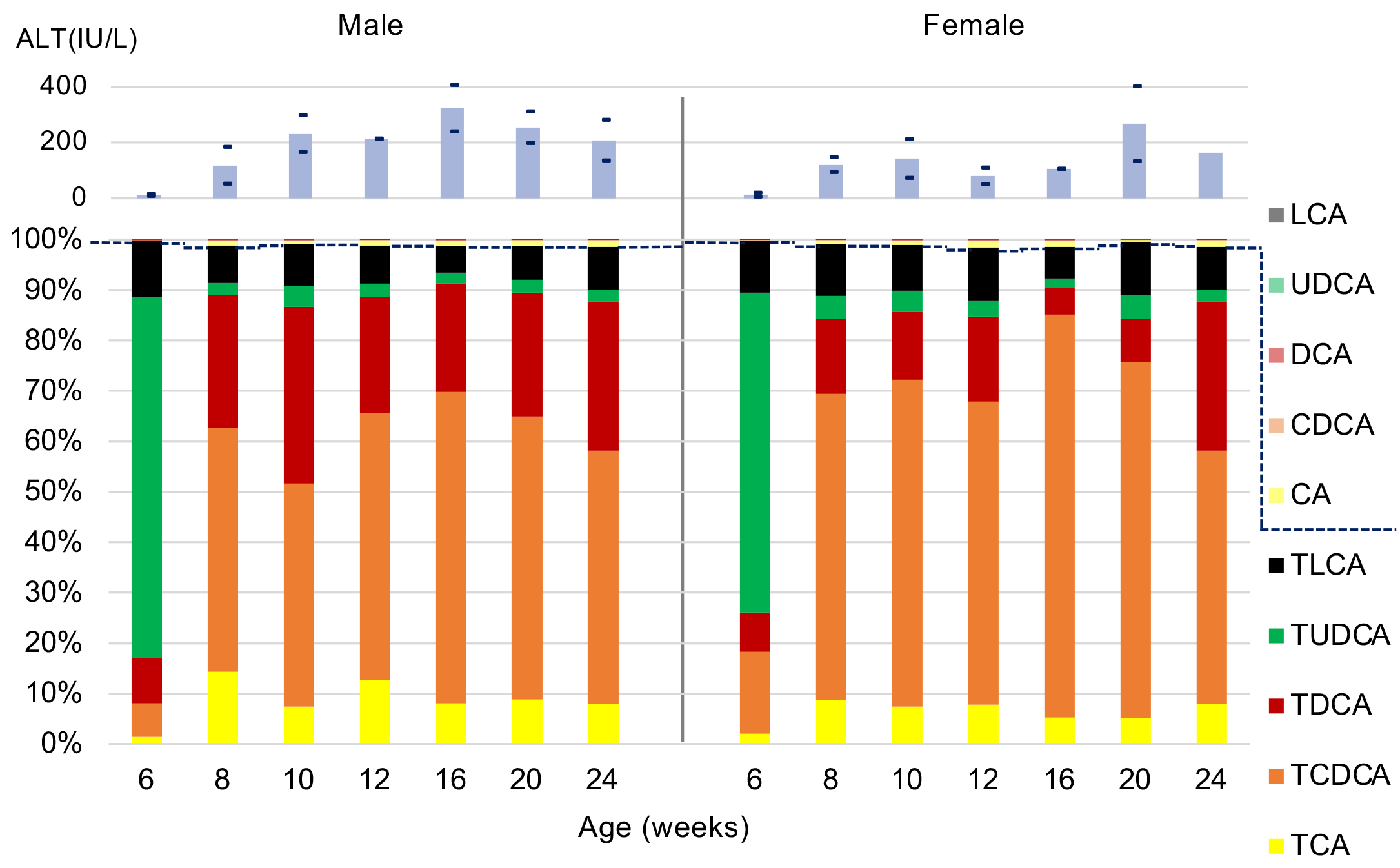

Supplement: S2 Fig — DKO mice were bred under UDCA administration, and offspring in both sexes stopped receiving UDCA at 6 weeks old. The changes in biliary BA compositions and serum ALT activities after discontinuation of UDCA are shown. Data represent the means of duplicate determinations. The broken line indicates the boundary between conjugated and unconjugated BAs. (TIF) [file pone.0271308.s003.tif]

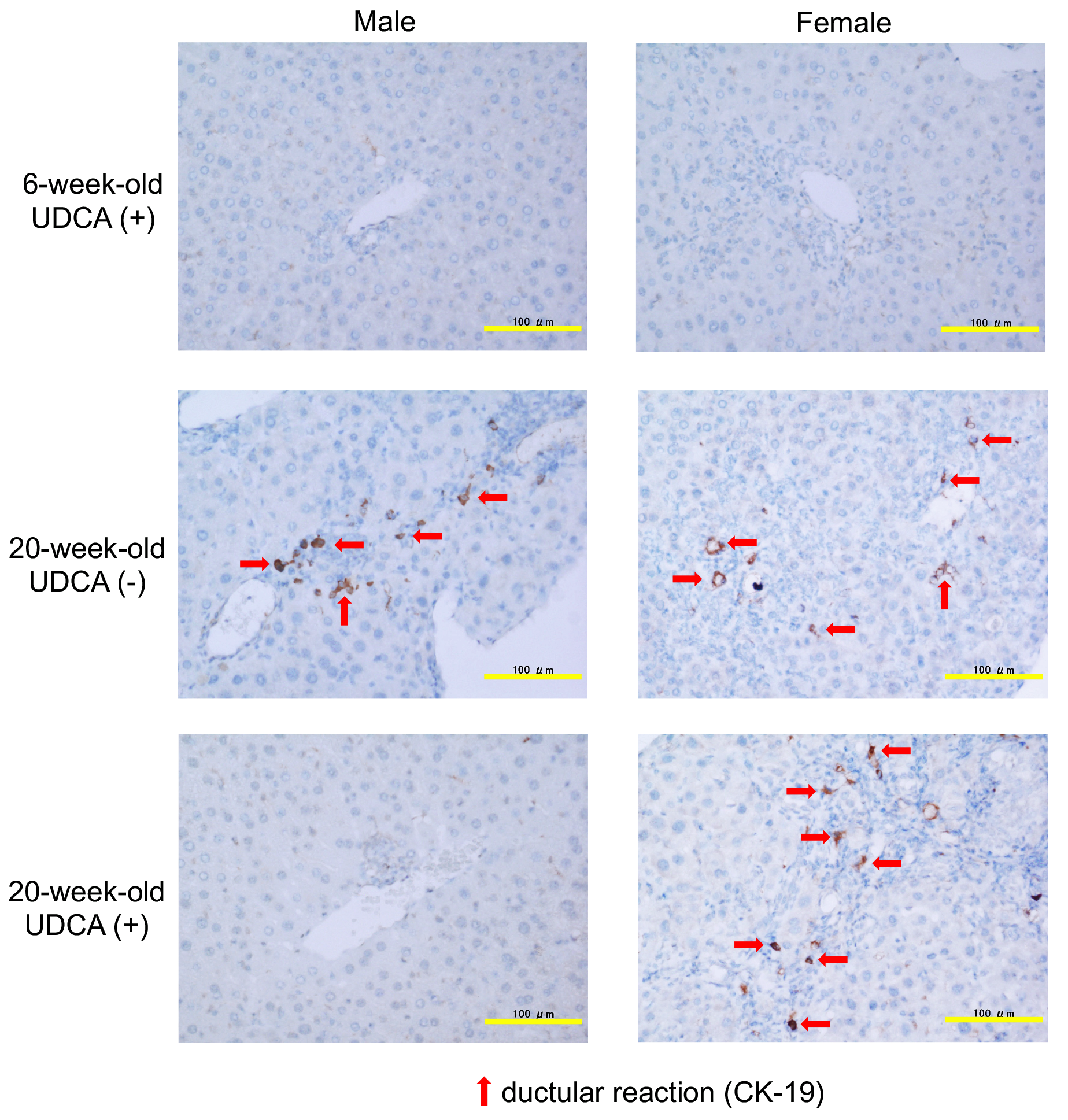

Supplement: S3 Fig — Expression of CK19 in the livers of DKO mice at the age of 6 weeks with (+) UDCA and 20 weeks with (+) or without (-) UDCA treatment. Red arrows indicate ductular reaction by immunohistochemical staining CK19. Scale bars, 100 μm. (TIF) [file pone.0271308.s004.tif]

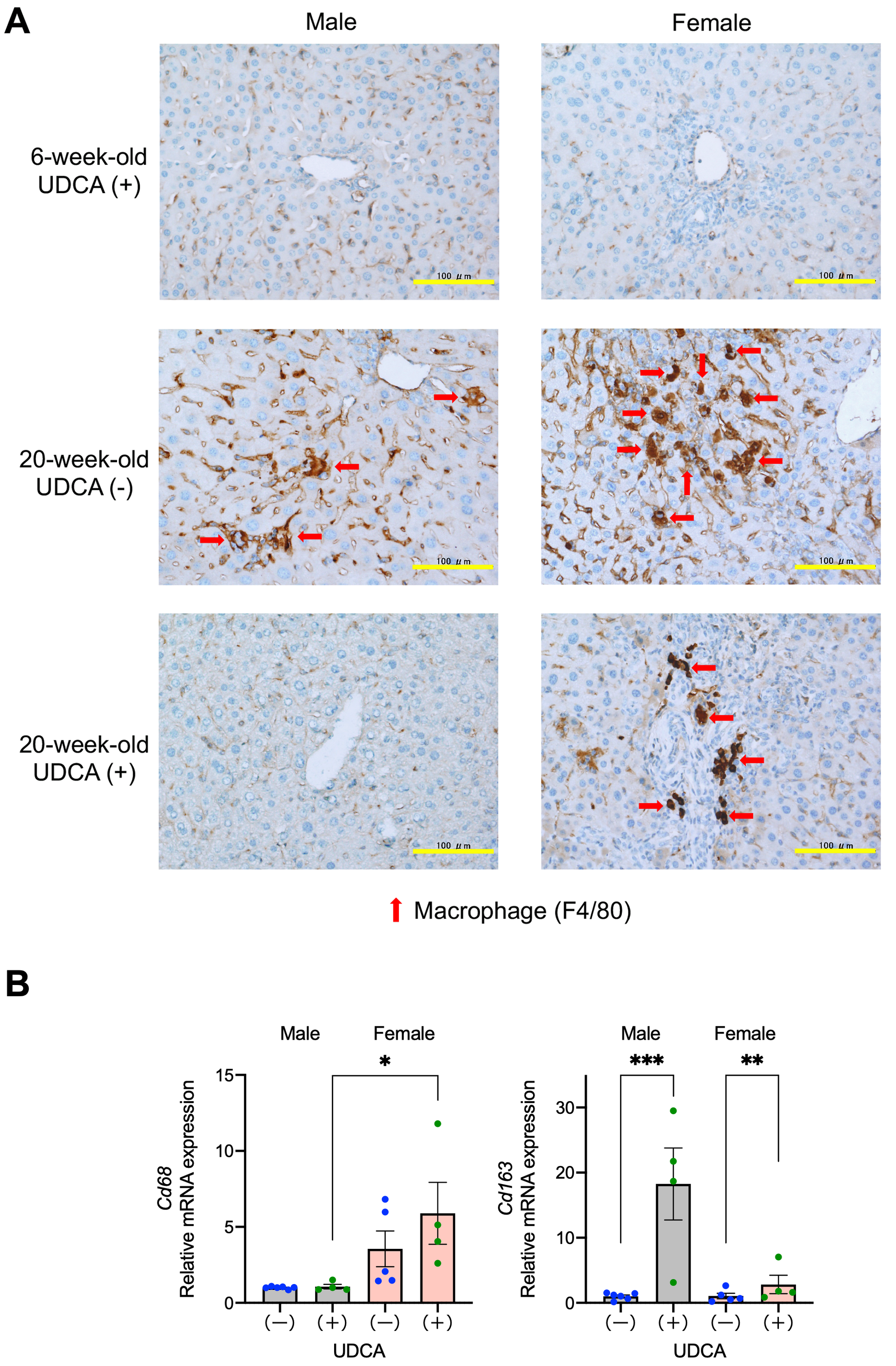

Supplement: S4 Fig — (A) Expression of F4/80 in the livers of DKO mice at the age of 6 weeks with (+) UDCA and 20 weeks with (+) or without (-) UDCA treatment. Red arrows indicate infiltration of macrophages by immunohistochemical staining F4/80. Scale bars, 100 μm. (B) Hepatic mRNA expression levels of Cd68 and Cd163 in DKO mice at 20 weeks with or without UDCA treatment. Each column and error bar represents the mean and SEM. *P < 0.05, **P < 0.01, and ***P < 0.001 were considered statistically significantly different by the Tukey-Kramer test. (TIF) [file pone.0271308.s005.tif]

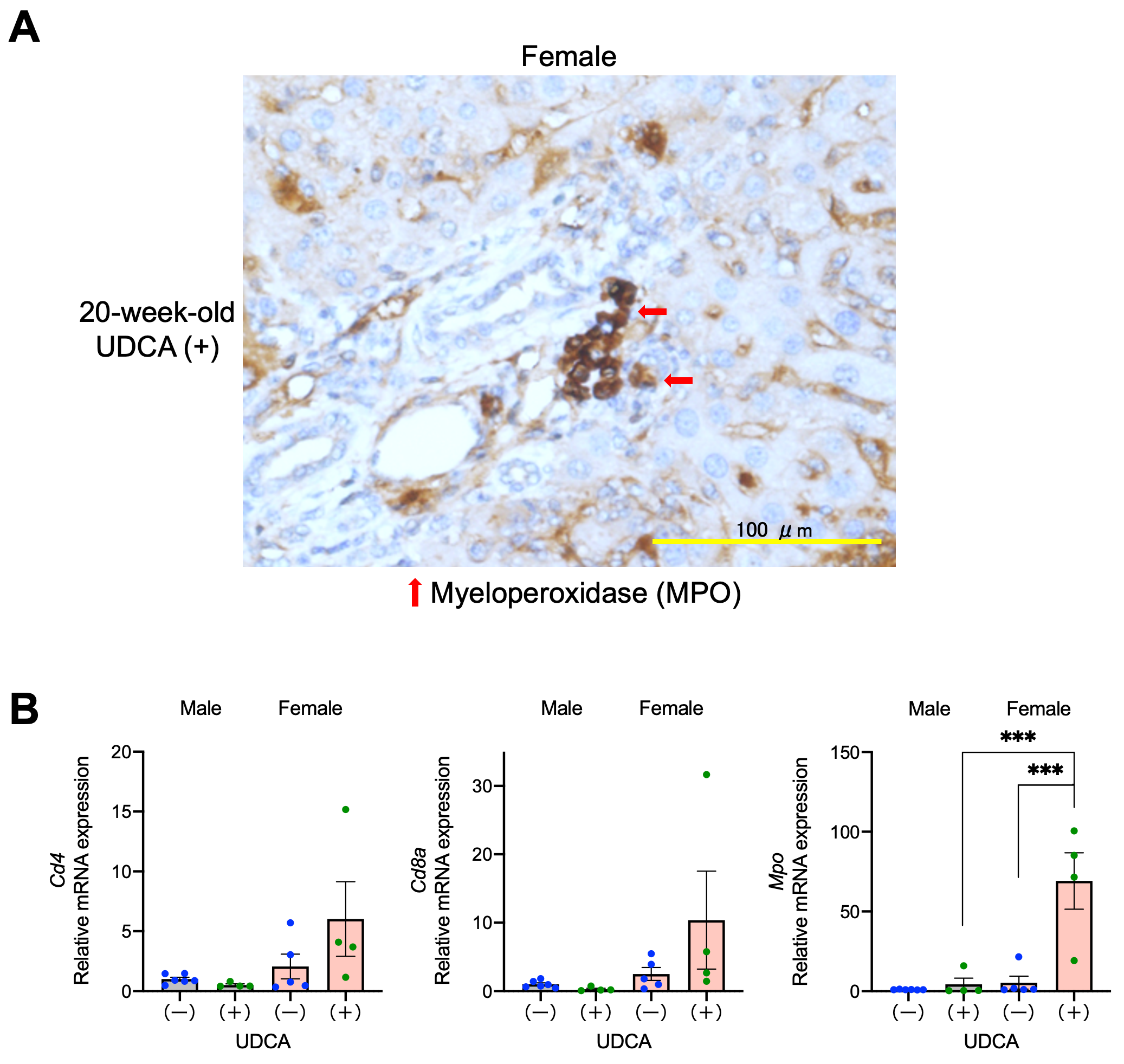

Supplement: S5 Fig — (A) Expression of MPO in the liver of female DKO mice at the age of 20 weeks with (+) UDCA treatment. Red arrows indicate infiltration of neutrophils by immunohistochemical staining MPO. Scale bars, 100 μm. (B) Hepatic mRNA expression levels of Cd4, Cd8a, and Mpo in DKO mice at 20 weeks with (+) or without (-) UDCA treatment. Each column and error bar represents the mean and SEM. ***P < 0.001 was considered statistically significantly different by the Tukey-Kramer test. (TIF) [file pone.0271308.s006.tif]

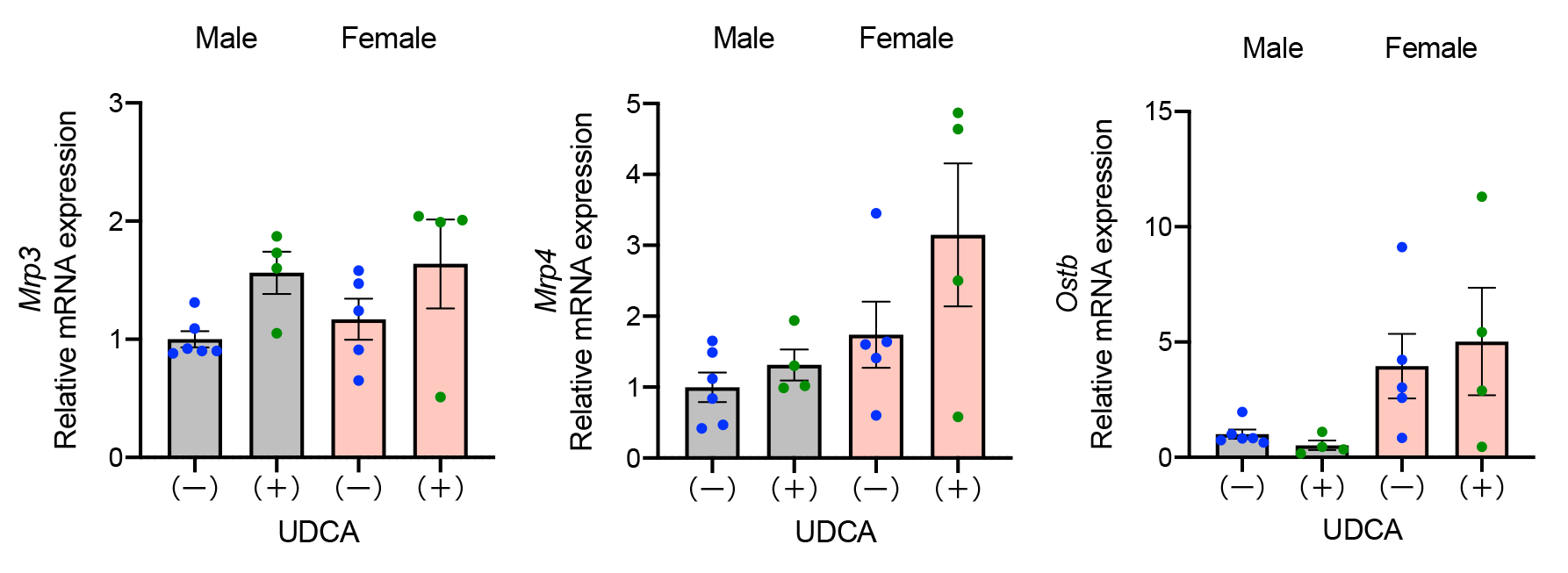

Supplement: S6 Fig — mRNA expression levels of Mrp3, Mrp4, and Ostb in the liver of DKO mice at the age of 20 weeks with (+) or without (-) UDCA treatment. Each column and error bar represents the mean and SEM. (TIF) [file pone.0271308.s007.tif]
