# Supplementary material for: Sex-, age-, and organ-dependent improvement of bile acid hydrophobicity by ursodeoxycholic acid treatment: A study using a mouse model with human-like bile acid composition
Source: PLoS One. 2022 Jul 12;17(7):e0271308. doi: 10.1371/journal.pone.0271308 (PMC9275687; doi:10.1371/journal.pone.0271308)
Supplement: S1 Table — (DOCX) [file pone.0271308.s008.docx]

**S1 Table. Sequences of oligonucleotide primers for qRT-PCR.**

| mRNA | Genbank  Accession No. | Forward | Reverse | Amplicon  Length  (bp) |
| --- | --- | --- | --- | --- |
| *Cyp7a1*  *Cyp8b1*  *Cyp27a1*  *Hmgcr*  *Fxr*  *Shp*  *Bsep*  *Ostb*  *Mrp3*  *Mrp4*  *Pxr*  *Car*  *Vdr*  *Hnf4a*  *Cyp3a11*  *Cyp2b10*  *Sult2a1*  *Lxra*  *Srebp1*  *Abcg5*  *Abcg8*  *Il1b*  *Tnfa*  *Ccl2*  *Tgfb1*  *Cd68*  *Cd163*  *Cd4*  *Cd8a*  *Mpo*  *Gapdh* | NM_007824  NM_010012  NM_024264  NM_008255  NM_001163700  NM_011850  NM_021022  NM_178933  NM_001363187  NM_001033336  NM_010936  NM_001243062  NM_009504  NM_008261  NM_007818  NM_009999  NM_001111296  NM_013839  NM_0011480  NM_031884  NM_026180  NM_008361  NM_001278601  NM_011333  NM_011577  NM_001291058  NM_001170395  NM_013488  NM_001081110  NM_010824  NM_008084 | 5’-AAGAGCAACTAAACAACCTG-3’  5’-TCCTGAGCTTATTCGGCTACA-3’  5’-CTTCCTGCTGACCAATGAAT-3’  5’-TGCCATCGATAGAGATAGGAA-3’  5’-GGTCATGCAGACCTGTTGGAA-3’  5’-CAAGGAGTATGCGTACCTGA-3’  5’-AGCAGGCTCAGCTGCATGAC-3’  5’-AACATGGACCACAGTGCAGAGA-3’  5’-CGGCTCAACACAATCATGGAC-3’  5’-TGGACCATCCGGGCTTACA-3’  5’-CCTACATGTTCAAGGGCGTCATC-3’  5’-TCCACGCCCTGACTTGTGA-3’  5’-TGGACATTGGCATGATGAAGG-3’  5’-ATGCCTGCCTCAAAGCCATC-3’  5’-ggcagcattgatccttatg-3’  5’-TGCTGTCGTTGAGCCAACC-3’  5’-GGGTAACACAAACCTTGTGAAGA -3’  5’-TGGAGACGTCACGGAGGTACA-3’  5’-CCATTGACAAGGCCATGC-3’  5’-GGCATGCTCAATGCTGTGAA-3’  5’-GGCTCAGGATCGGCTTTCAC-3’  5’-TCCAGGATGAGGACATGAGCAC-3’  5’-ACTCCAGGCGGTGCCTATGT-3’  5’-CCACTCACCTGCTGCTACTCAT-3’  5’-TGGAGCAACATGTGGAACTC-3’  5’-AGCATAGTTCTTTCTCCAGC-3’  5’-GTGGACTCTGAAGCGACGACA-3’  5’-CTAGTTCCAGGCCCTCGGTA-3’  5’-AACCACCACTGTGAAATTCCTGTAG-3’  5’-AAGCGCCTGAATCCTCGATG-3’  5’-ATCAAGAAGGTGGTGAAGCA-3’ | 5’-TTCCCACTTTCATCAAGGTA-3’  5’-ATCGACGGAACTTCCTGAAC-3’  5’-AGCTTTTAGCAGAGGCATGT-3’  5’-GCCATCACAGTGCCACATA-3’  5’-TGACGATCGCTGTGAGCAGA-3’  5’-ATCTCTTCTTCCGCCCTATC-3’  5’-AATGGCCCGAGCAATAGCAA-3’  5’-GCTTGTCATGACCACCAGGA-3’  5’-CCGCAATGAGGTTGACTGGA-3’  5’-GATGTCGTCAGGAACAAGAACCAA-3’  5’-TGTCGAACATCGTGTTGAACCTC-3’  5’-CTTCCAGCAAACGGACAGATG-3’  5’-GATGGCGATAATGTGCTGTTG-3’  5’-ATCTTGCCCGGGTCACTCA-3’  5’-aagaactccttgagggagac-3’  5’-CCACTAAACATTGGGCTTCCT-3’  5’-GCTCAAACCATGATCCGAATAGA-3’  5’-CAGCTCATTCATGGCTCTGGA-3’  5’-GGTCATGTTGGAAACCACGC-3’  5’-ATGATACAGGCCATCCTGACTCTC-3’  5’- CCTTGACACAGGCATGAAGCA-3’  5’-GAACGTCACACACCAGCAGGTTA-3’  5’-GTGAGGGTCTGGGCCATAGAA-3’  5’-TGGTGATCCTCTTGTAGCTCTCC-3’  5’-GTCAGCAGCCGGTTACCA-3’  5’-ATGATGAGAGGCAGCAAGAG-3’  5’-AAGTCCAGATCATCCGCCTTTG-3’  5’-GGGTGAGAACAGCAGTGATCAA-3’  5’-TGCCTGAGTCGTATCTGTCAAACC-3’  5’-GCAGGTAGTCCCGGTATGTGATG-3’  5’-CTGTAGCCGTATTCATTGTCA-3’ | 244  81  229  154  142  232  122  150  91  101  138  88  149  67  260  161  95  149  162  78  82  105  160  76  73  137  114  136  87  100  191 |
