# Supplementary material for: Sex-, age-, and organ-dependent improvement of bile acid hydrophobicity by ursodeoxycholic acid treatment: A study using a mouse model with human-like bile acid composition
Source: PLoS One. 2022 Jul 12;17(7):e0271308. doi: 10.1371/journal.pone.0271308 (PMC9275687; doi:10.1371/journal.pone.0271308)
Supplement: S2 Table — (DOCX) [file pone.0271308.s009.docx]

**S2 Table. Effects of UDCA treatment on BA composition of total BA pool.**

| Total BA pool | Male | | Female | |
| --- | --- | --- | --- | --- |
|  | UDCA (–) | UDCA (+) | UDCA (–) | UDCA (+) |
|  | n = 10 | n = 4 | n = 4 | n = 4 |
| TCA (%) | 4.8 ± 0.6 | 0.5 ± 0.2^a^ | 3.3 ± 0.2^b^ | 0.5 ± 0.1^a^ |
| TCDCA (%) | 34.6 ± 1.6 | 4.6 ± 1.4^a^ | 50.5 ± 4.8^ab^ | 8.5 ± 1.0^ac^ |
| TDCA (%) | 24.4 ± 2.4 | 5.5 ± 0.3^a^ | 12.8 ± 1.4^a^ | 8.7 ± 1.2^a^ |
| TUDCA (%) | 1.1 ± 0.2 | 67.3 ± 1.9^a^ | 1.9 ± 0.2^b^ | 43.7 ± 5.9^abc^ |
| TLCA (%) | 10.5 ± 0.7 | 18.7 ± 0.7^a^ | 15.6 ± 1.1 | 27.2 ± 3.6^abc^ |
| CA (%) | 5.9 ± 0.8 | 0.1 ± 0.0^a^ | 2.4 ± 0.5^a^ | 0.6 ± 0.2^a^ |
| CDCA (%) | 11.2 ± 3.0 | 0.1 ± 0.0 | 9.4 ± 3.0 | 1.5 ± 0.7 |
| DCA (%) | 5.0 ± 0.7 | 0.2 ± 0.0^a^ | 1.9 ± 0.9^a^ | 1.3 ± 0.4^a^ |
| UDCA (%) | 0.2 ± 0.0 | 2.7 ± 0.3^a^ | 0.2 ± 0.1 | 5.9 ± 1.6^abc^ |
| LCA (%) | 2.2 ± 0.5 | 0.3 ± 0.0 | 1.9 ± 0.6 | 2.2 ± 0.8 |

DKO mice at 20 weeks of age were compared. Each data represents the mean and SEM.

UDCA (–), without UDCA; UDCA (+), with UDCA.

^a^p<0.05, significantly different from Male UDCA (–) by Tukey-Kramer test.

^b^p<0.05, significantly different from Male UDCA (+) by Tukey-Kramer test.

^c^p<0.05, significantly different from Female UDCA (–) by Tukey-Kramer test.
