# Supplementary material for: Sex-, age-, and organ-dependent improvement of bile acid hydrophobicity by ursodeoxycholic acid treatment: A study using a mouse model with human-like bile acid composition
Source: PLoS One. 2022 Jul 12;17(7):e0271308. doi: 10.1371/journal.pone.0271308 (PMC9275687; doi:10.1371/journal.pone.0271308)
Supplement: S3 Table — (DOCX) [file pone.0271308.s010.docx]

**S3 Table. Effects of UDCA treatment on BA concentration of total BA pool.**

| Total BA pool | Male | | Female | |
| --- | --- | --- | --- | --- |
|  | UDCA (–) | UDCA (+) | UDCA (–) | UDCA (+) |
|  | n = 10 | n = 4 | n = 4 | n = 4 |
| TCA (µmol/100 g BW) | 1.5 ± 0.3 | 0.2 ± 0.1^a^ | 1.9 ± 0.2^b^ | 0.3 ± 0.1^ac^ |
| TCDCA (µmol/100 g BW) | 10.9 ± 0.9 | 2.1 ± 0.5^a^ | 29.3 ± 4.1^ab^ | 6.3 ± 0.9^c^ |
| TDCA (µmol/100 g BW) | 7.8 ± 0.9 | 2.5 ± 0.1^a^ | 7.2 ± 0.7 | 6.9 ± 1.7 |
| TUDCA (µmol/100 g BW) | 0.4 ± 0.2 | 31.4 ± 2.2^a^ | 1.1 ± 0.2^b^ | 35.8 ± 8.6^ac^ |
| TLCA (µmol/100 g BW) | 3.3 ± 0.3 | 8.7 ± 0.5^a^ | 9.0 ± 1.1^a^ | 19.7 ± 2.3^abc^ |
| CA (µmol/100 g BW) | 1.8 ± 0.3 | 0.0 ± 0.0^a^ | 1.3 ± 0.3 | 0.4 ± 0.1^a^ |
| CDCA (µmol/100 g BW) | 3.3 ± 0.7 | 0.0 ± 0.0 | 5.2 ± 1.4^b^ | 1.0 ± 0.4^c^ |
| DCA (µmol/100 g BW) | 1.5 ± 0.2 | 0.1 ± 1.3^a^ | 1.0 ± 0.5 | 0.9 ± 0.2 |
| UDCA (µmol/100 g BW) | 0.1 ± 0.0 | 1.3 ± 0.2 | 0.1 ± 0.0 | 4.5 ± 1.5^abc^ |
| LCA (µmol/100 g BW) | 0.7 ± 0.2 | 0.2 ± 0.0 | 1.0 ± 0.3 | 1.6 ± 0.6^b^ |

DKO mice at 20 weeks of age were compared. Each data represents the mean and SEM.

BW, body weight.

UDCA (–), without UDCA; UDCA (+), with UDCA.

^a^p<0.05, significantly different from Male UDCA (–) by Tukey-Kramer test.

^b^p<0.05, significantly different from Male UDCA (+) by Tukey-Kramer test.

^c^p<0.05, significantly different from Female UDCA (–) by Tukey-Kramer test.
