# Supplementary material for: Sex-, age-, and organ-dependent improvement of bile acid hydrophobicity by ursodeoxycholic acid treatment: A study using a mouse model with human-like bile acid composition
Source: PLoS One. 2022 Jul 12;17(7):e0271308. doi: 10.1371/journal.pone.0271308 (PMC9275687; doi:10.1371/journal.pone.0271308)
Supplement: S4 Table — (DOCX) [file pone.0271308.s011.docx]

**S4 Table. Effects of UDCA treatment on fecal BA composition.**

| Fecal BA | Male | | Female | |
| --- | --- | --- | --- | --- |
|  | UDCA (–) | UDCA (+) | UDCA (–) | UDCA (+) |
|  | n = 10 | n = 4 | n = 3 | n = 4 |
| TCA (%) | 0.1 ± 0.1 | 0.0 ± 0.0 | 0.0 ± 0.0 | 0.0 ± 0.0 |
| TCDCA (%) | 0.9 ± 0.2 | 0.1 ± 0.0 | 0.4 ± 0.1 | 0.3 ± 0.2 |
| TDCA (%) | 0.2 ± 0.1 | 0.1 ± 0.0 | 0.1 ± 0.1 | 0.2 ± 0.1 |
| TUDCA (%) | 0.0 ± 0.0 | 6.1 ± 0.8^a^ | 0.0 ± 0.0^b^ | 1.5 ± 0.4^ab^ |
| TLCA (%) | 0.3 ± 0.1 | 11.4 ± 5.4^a^ | 0.7 ± 0.4^b^ | 3.0 ± 1.2 |
| CA (%) | 1.9 ± 0.2 | 0.8 ± 0.2^a^ | 1.5 ± 0.5 | 0.4 ± 0.2^a^ |
| CDCA (%) | 3.0 ± 0.6 | 0.6 ± 0.3 | 3.1 ± 1.4 | 1.4 ± 0.8 |
| DCA (%) | 17.3 ± 1.5 | 1.7 ± 0.5^a^ | 8.2 ± 1.5^a^ | 3.0 ± 0.6^a^ |
| UDCA (%) | 0.8 ± 0.1 | 17.1 ± 6.9^a^ | 0.7 ± 0.1^b^ | 9.4 ± 3.1 |
| LCA (%) | 75.4 ± 1.9 | 62.0 ± 13.1 | 85.3 ± 1.2 | 80.8 ± 6.0 |

DKO mice at 20 weeks of age were compared. Each data represents the mean and SEM.

UDCA (–), without UDCA; UDCA (+), with UDCA.

^a^p<0.05, significantly different from Male UDCA (–) by Tukey-Kramer test.

^b^p<0.05, significantly different from Male UDCA (+) by Tukey-Kramer test.

^c^p<0.05, significantly different from Female UDCA (–) by Tukey-Kramer test.
