# Supplementary material for: Sex-, age-, and organ-dependent improvement of bile acid hydrophobicity by ursodeoxycholic acid treatment: A study using a mouse model with human-like bile acid composition
Source: PLoS One. 2022 Jul 12;17(7):e0271308. doi: 10.1371/journal.pone.0271308 (PMC9275687; doi:10.1371/journal.pone.0271308)
Supplement: S5 Table — (DOCX) [file pone.0271308.s012.docx]

**S5 Table. Effects of UDCA treatment on fecal BA concentration.**

| Fecal BA | Male | | Female | |
| --- | --- | --- | --- | --- |
|  | UDCA (–) | UDCA (+) | UDCA (–) | UDCA (+) |
|  | n = 10 | n = 4 | n = 3 | n = 4 |
| TCA (nmol/g wet feces) | 0.6 ± 0.5 | 3.0 ± 1.8 | 0.1 ± 0.0 | 0.2 ± 0.1 |
| TCDCA (nmol/g wet feces) | 7.2 ± 2.5 | 6.9 ± 4.4 | 3.0 ± 1.5 | 9.8 ± 4.1 |
| TDCA (nmol/g wet feces) | 1.7 ± 0.5 | 8.8 ± 5.2 | 0.4 ± 0.1 | 6.4 ± 2.2 |
| TUDCA (nmol/g wet feces) | 0.1 ± 0.0 | 323.9 ± 108.2^a^ | 0.1 ± 0.1^b^ | 82.4 ± 21.3^b^ |
| TLCA (nmol/g wet feces) | 3.5 ± 1.6 | 820.2 ± 529.3^a^ | 4.0 ± 1.3 | 150.0 ± 29.8 |
| CA (nmol/g wet feces) | 14.6 ± 2.3 | 36.3 ± 14.6 | 9.4 ± 1.5 | 16.2 ± 3.6 |
| CDCA (nmol/g wet feces) | 25.4 ± 7.2 | 37.4 ± 26.9 | 17.9 ± 0.8 | 59.3 ± 26.8 |
| DCA (nmol/g wet feces) | 145.1 ± 25.6 | 98.6 ± 55.1 | 72.1 ± 28.6 | 165.9 ± 31.7 |
| UDCA (nmol/g wet feces) | 6.2 ± 1.4 | 1150.1 ± 751.8^a^ | 6.0 ± 2.1 | 570.5 ± 249.0 |
| LCA (nmol/g wet feces) | 635.6 ± 123.2 | 2712.2 ± 625.4 | 682.6 ± 215.0 | 5239.6 ± 1512.1^ac^ |

DKO mice at 20 weeks of age were compared. Each data represents the mean and SEM.

UDCA (–), without UDCA; UDCA (+), with UDCA.

^a^p<0.05, significantly different from Male UDCA (–) by Tukey-Kramer test.

^b^p<0.05, significantly different from Male UDCA (+) by Tukey-Kramer test.

^c^p<0.05, significantly different from Female UDCA (–) by Tukey-Kramer test.
