# Supplementary material for: Sex-, age-, and organ-dependent improvement of bile acid hydrophobicity by ursodeoxycholic acid treatment: A study using a mouse model with human-like bile acid composition
Source: PLoS One. 2022 Jul 12;17(7):e0271308. doi: 10.1371/journal.pone.0271308 (PMC9275687; doi:10.1371/journal.pone.0271308)
Supplement: S6 Table — (DOCX) [file pone.0271308.s013.docx]

**S6 Table. Effects of UDCA treatment on hepatic BA composition.**

| Liver BA | Male | | Female | |
| --- | --- | --- | --- | --- |
|  | UDCA (–) | UDCA (+) | UDCA (–) | UDCA (+) |
|  | n = 6 | n = 4 | n = 5 | n = 4 |
| TCA (%) | 5.4 ± 0.4 | 0.5 ± 0.2^a^ | 2.8 ± 0.8^ab^ | 0.3 ± 0.1^ac^ |
| TCDCA (%) | 36.9 ± 2.5 | 3.3 ± 1.3^a^ | 58.5 ± 4.5^ab^ | 5.6 ± 1.1^ac^ |
| TDCA (%) | 27.0 ± 1.8 | 5.0 ± 0.5^a^ | 6.3 ± 1.3^a^ | 6.8 ± 0.4^a^ |
| TUDCA (%) | 0.6 ± 0.1 | 61.2 ± 3.0^a^ | 1.7 ± 0.3^b^ | 27.3 ± 5.2^abc^ |
| TLCA (%) | 8.4 ± 0.6 | 26.0 ± 2.7^a^ | 14.0 ± 2.9 | 50.9 ± 9.2^abc^ |
| CA (%) | 1.3 ± 0.2 | 0.1 ± 0.0^a^ | 0.7 ± 0.2^b^ | 0.1 ± 0.1^a^ |
| CDCA (%) | 17.3 ± 1.5 | 0.1 ± 0.0^a^ | 13.3 ± 2.7^b^ | 0.5 ± 0.34^ac^ |
| DCA (%) | 0.4 ± 0.1 | 0.1 ± 0.0^a^ | 0.1 ± 0.0^a^ | 0.1 ± 0.0^a^ |
| UDCA (%) | 0.9 ± 0.1 | 3.2 ± 0.3 | 1.0 ± 0.2 | 6.9 ± 4.2 |
| LCA (%) | 1.8 ± 0.1 | 0.5 ± 0.1^a^ | 1.5 ± 0.3 | 1.5 ± 0.6 |

DKO mice at 20 weeks of age were compared. Each data represents the mean and SEM.

UDCA (–), without UDCA; UDCA (+), with UDCA.

^a^p<0.05, significantly different from Male UDCA (–) by Tukey-Kramer test.

^b^p<0.05, significantly different from Male UDCA (+) by Tukey-Kramer test.

^c^p<0.05, significantly different from Female UDCA (–) by Tukey-Kramer test.
