# Supplementary material for: Sex-, age-, and organ-dependent improvement of bile acid hydrophobicity by ursodeoxycholic acid treatment: A study using a mouse model with human-like bile acid composition
Source: PLoS One. 2022 Jul 12;17(7):e0271308. doi: 10.1371/journal.pone.0271308 (PMC9275687; doi:10.1371/journal.pone.0271308)
Supplement: S7 Table — (DOCX) [file pone.0271308.s014.docx]

**S7 Table. Effects of UDCA treatment on hepatic BA concentration.**

| Liver BA | Male | | Female | |
| --- | --- | --- | --- | --- |
|  | UDCA (–) | UDCA (+) | UDCA (–) | UDCA (+) |
|  | n = 6 | n = 4 | n = 5 | n = 4 |
| TCA (nmol/g) | 14.3 ± 1.4 | 1.3 ± 0.5^a^ | 10.2 ± 3.6 | 2.8 ± 1.2^a^ |
| TCDCA (nmol/g) | 96.8 ± 9.7 | 8.5 ± 3.4 | 203.8 ± 36.9^ab^ | 52.1 ± 24.3^c^ |
| TDCA (nmol/g) | 70.6 ± 6.5 | 12.7 ± 1.5^a^ | 21.1 ± 5.7^a^ | 57.1 ± 14.8^bc^ |
| TUDCA (nmol/g) | 1.5 ± 0.3 | 156.1 ± 14.7^a^ | 5.4 ± 0.8^b^ | 211.0 ± 54.7^ac^ |
| TLCA (nmol/g) | 21.8 ± 1.8 | 66.6 ± 10.2 | 45.5 ± 6.9 | 524.4 ± 210.0^abc^ |
| CA (nmol/g) | 3.6 ± 0.7 | 0.1 ± 0.0^a^ | 2.7 ± 0.8 | 0.6 ± 0.1^a^ |
| CDCA (nmol/g) | 46.7 ± 7.6 | 0.2 ± 0.0^a^ | 44.9 ± 10.3^b^ | 2.4 ± 0.6^ac^ |
| DCA (nmol/g) | 1.1 ± 0.3 | 0.2 ± 0.0^a^ | 0.3 ± 0.1^a^ | 0.3 ± 0.0^a^ |
| UDCA (nmol/g) | 2.4 ± 0.2 | 8.1 ± 0.6 | 3.2 ± 0.9 | 30.4 ± 10.5^abc^ |
| LCA (nmol/g) | 4.9 ± 0.6 | 1.3 ± 0.1^a^ | 4.8 ± 0.8 | 8.6 ± 1.6^abc^ |

DKO mice at 20 weeks of age were compared. Each data represents the mean and SEM.

UDCA (–), without UDCA; UDCA (+), with UDCA.

^a^p<0.05, significantly different from Male UDCA (–) by Tukey-Kramer test.

^b^p<0.05, significantly different from Male UDCA (+) by Tukey-Kramer test.

^c^p<0.05, significantly different from Female UDCA (–) by Tukey-Kramer test.
