# Supplementary material for: Sex-, age-, and organ-dependent improvement of bile acid hydrophobicity by ursodeoxycholic acid treatment: A study using a mouse model with human-like bile acid composition
Source: PLoS One. 2022 Jul 12;17(7):e0271308. doi: 10.1371/journal.pone.0271308 (PMC9275687; doi:10.1371/journal.pone.0271308)
Supplement: S8 Table — (DOCX) [file pone.0271308.s015.docx]

**S8 Table. Effects of UDCA treatment on biliary BA composition.**

| Gallbladder BA | Male | | Female | |
| --- | --- | --- | --- | --- |
|  | UDCA (–) | UDCA (+) | UDCA (–) | UDCA (+) |
|  | n = 6 | n = 4 | n = 5 | n = 4 |
| TCA (%) | 8.3 ± 0.4 | 0.5 ± 0.2^a^ | 5.0 ± 0.6^ab^ | 0.8 ± 0.2^ac^ |
| TCDCA (%) | 56.1 ± 2.7 | 4.8 ± 1.5^a^ | 75.0 ± 3.1^ab^ | 11.0 ± 1.5^ac^ |
| TDCA (%) | 25.1 ± 2.2 | 5.6 ± 0.4^a^ | 7.0 ± 1.4^a^ | 10.8 ± 1.1^a^ |
| TUDCA (%) | 2.3 ± 0.2 | 70.9 ± 2.6^a^ | 3.3 ± 0.8^b^ | 58.0 ± 1.1^abc^ |
| TLCA (%) | 6.9 ± 0.7 | 18.1 ± 1.1^a^ | 8.7 ± 1.1^b^ | 19.2 ± 1.2^ac^ |
| CA (%) | 1.1 ± 0.0 | 0.0 ± 0.0^a^ | 0.8 ± 0.2^b^ | 0.1 ± 0.0^ac^ |
| CDCA (%) | 0.2 ± 0.0 | 0.0 ± 0.0^a^ | 0.2 ± 0.0^b^ | 0.0 ± 0.0^ac^ |
| DCA (%) | 0.1 ± 0.0 | 0.0 ± 0.0^a^ | 0.0 ± 0.0 | 0.0 ± 0.0^a^ |
| UDCA (%) | 0.0 ± 0.0 | 0.0 ± 0.0 | 0.0 ± 0.0 | 0.1 ± 0.0^ac^ |
| LCA (%) | 0.0 ± 0.0 | 0.0 ± 0.0 | 0.0 ± 0.0 | 0.0 ± 0.0 |

DKO mice at 20 weeks of age were compared. Each data represents the mean and SEM.

UDCA (–), without UDCA; UDCA (+), with UDCA.

^a^p<0.05, significantly different from Male UDCA (–) by Tukey-Kramer test.

^b^p<0.05, significantly different from Male UDCA (+) by Tukey-Kramer test.

^c^p<0.05, significantly different from Female UDCA (–) by Tukey-Kramer test.
