# Supplementary material for: Sex-, age-, and organ-dependent improvement of bile acid hydrophobicity by ursodeoxycholic acid treatment: A study using a mouse model with human-like bile acid composition
Source: PLoS One. 2022 Jul 12;17(7):e0271308. doi: 10.1371/journal.pone.0271308 (PMC9275687; doi:10.1371/journal.pone.0271308)
Supplement: S9 Table — (DOCX) [file pone.0271308.s016.docx]

**S9 Table. Effects of UDCA treatment on biliary BA concentration.**

| Gallbladder BA | Male | | Female | |
| --- | --- | --- | --- | --- |
|  | UDCA (–) | UDCA (+) | UDCA (–) | UDCA (+) |
|  | n = 6 | n = 4 | n = 5 | n = 4 |
| TCA (nmol/whole) | 25.5 ± 11.4 | 12.6 ± 5.9 | 31.7 ± 16.6 | 35.4 ± 12.8 |
| TCDCA (nmol/whole) | 153.8 ± 53.8 | 129.7 ± 57.6 | 403.5 ± 175.0 | 622.7 ± 226.6 |
| TDCA (nmol/whole) | 88.2 ± 47.7 | 144.1 ± 41.6 | 45.2 ± 21.2 | 684.5 ± 261.7^abc^ |
| TUDCA (nmol/whole) | 7.1 ± 2.9 | 1701.9 ± 382.9 | 20.9 ± 9.5 | 3417.6 ± 1102.0^ac^ |
| TLCA (nmol/whole) | 23.3 ± 12.1 | 464.4 ± 135.8 | 59.4 ± 30.3 | 1209.6 ± 400.2^ac^ |
| CA (nmol/whole) | 3.2 ± 1.3 | 0.4 ± 0.2 | 4.0 ± 1.7 | 4.6 ± 2.2 |
| CDCA (nmol/whole) | 0.4 ± 0.1 | 0.0 ± 0.0 | 0.8 ± 0.5 | 0.4 ± 0.2 |
| DCA (nmol/whole) | 0.2 ± 0.1 | 0.1 ± 0.0 | 0.1 ± 0.0 | 0.3 ± 0.1 |
| UDCA (nmol/whole) | 0.0 ± 0.0 | 0.8 ± 0.3 | 0.0 ± 0.0 | 4.8 ± 2.3^ac^ |
| LCA (nmol/whole) | 0.1 ± 0.0 | 0.1 ± 0.0 | 0.1 ± 0.0 | 0.2 ± 0.0^ac^ |

DKO mice at 20 weeks of age were compared. Each data represents the mean and SEM.

UDCA (–), without UDCA; UDCA (+), with UDCA.

^a^p<0.05, significantly different from Male UDCA (–) by Tukey-Kramer test.

^b^p<0.05, significantly different from Male UDCA (+) by Tukey-Kramer test.

^c^p<0.05, significantly different from Female UDCA (–) by Tukey-Kramer test.
