# Supplementary material for: Sex-, age-, and organ-dependent improvement of bile acid hydrophobicity by ursodeoxycholic acid treatment: A study using a mouse model with human-like bile acid composition
Source: PLoS One. 2022 Jul 12;17(7):e0271308. doi: 10.1371/journal.pone.0271308 (PMC9275687; doi:10.1371/journal.pone.0271308)
Supplement: S10 Table — (DOCX) [file pone.0271308.s017.docx]

**S10 Table. Effects of UDCA treatment on serum BA composition.**

| Serum BA | Male | | Female | |
| --- | --- | --- | --- | --- |
|  | UDCA (–) | UDCA (+) | UDCA (–) | UDCA (+) |
|  | n = 6 | n = 4 | n = 5 | n = 4 |
| TCA (%) | 0.1 ± 0.0 | 0.2 ± 0.2 | 0.1 ± 0.1 | 0.3 ± 0.3 |
| TCDCA (%) | 2.2 ± 0.7 | 2.2 ± 1.4 | 12.4 ± 3.3^ab^ | 3.8 ± 2.5 |
| TDCA (%) | 1.9 ± 0.6 | 3.2 ± 0.8 | 1.0 ± 0.4 | 4.9 ± 2.2 |
| TUDCA (%) | 1.3 ± 1.3 | 61.5 ± 1.2^a^ | 0.4 ± 0.1^b^ | 26.7 ± 5.9^abc^ |
| TLCA (%) | 2.6 ± 1.6 | 15.7 ± 1.2^a^ | 3.5 ± 0.4^b^ | 16.0 ± 1.4^ac^ |
| CA (%) | 7.1 ± 0.6 | 2.1 ± 0.6^a^ | 5.1 ± 1.4 | 1.9 ± 0.5^a^ |
| CDCA (%) | 40.5 ± 2.5 | 0.5 ± 0.1^a^ | 54.7 ± 2.2^ab^ | 2.3 ± 0.8^ac^ |
| DCA (%) | 31.3 ± 3.2 | 1.2 ± 0.2^a^ | 8.6 ± 2.0^a^ | 5.5 ± 0.4^a^ |
| UDCA (%) | 1.8 ± 0.2 | 9.0 ± 1.0 | 3.4 ± 0.6 | 29.0 ± 6.9^abc^ |
| LCA (%) | 11.2 ± 0.5 | 4.5 ± 0.8 | 10.8 ± 1.4^a^ | 9.7 ± 3.3 |

DKO mice at 20 weeks of age were compared. Each data represents the mean and SEM.

UDCA (–), without UDCA; UDCA (+), with UDCA.

^a^p<0.05, significantly different from Male UDCA (–) by Tukey-Kramer test.

^b^p<0.05, significantly different from Male UDCA (+) by Tukey-Kramer test.

^c^p<0.05, significantly different from Female UDCA (–) by Tukey-Kramer test.
