# Supplementary material for: Sex-, age-, and organ-dependent improvement of bile acid hydrophobicity by ursodeoxycholic acid treatment: A study using a mouse model with human-like bile acid composition
Source: PLoS One. 2022 Jul 12;17(7):e0271308. doi: 10.1371/journal.pone.0271308 (PMC9275687; doi:10.1371/journal.pone.0271308)
Supplement: S11 Table — (DOCX) [file pone.0271308.s018.docx]

**S11 Table. Effects of UDCA treatment on serum BA concentration.**

| Serum BA | Male | | Female | |
| --- | --- | --- | --- | --- |
|  | UDCA (–) | UDCA (+) | UDCA (–) | UDCA (+) |
|  | n = 6 | n = 4 | n = 5 | n = 4 |
| TCA (µM) | 0.0 ± 0.0 | 0.3 ± 0.3 | 0.0 ± 0.0 | 0.4 ± 0.4 |
| TCDCA (µM) | 0.3 ± 0.1 | 2.5 ± 2.4 | 2.6 ± 1.3 | 4.3 ± 3.9 |
| TDCA (µM) | 0.2 ± 0.1 | 2.1 ± 1.7 | 0.2 ± 0.1 | 4.3 ± 3.6 |
| TUDCA (µM) | 0.2 ± 0.2 | 28.2 ± 19.8 | 0.1 ± 0.0 | 15.8 ± 10.7 |
| TLCA (µM) | 0.3 ± 0.2 | 8.4 ± 6.4 | 0.6 ± 0.2 | 8.9 ± 5.7 |
| CA (µM) | 0.7 ± 0.1 | 0.4 ± 0.1 | 0.7 ± 0.1 | 0.6 ± 0.3 |
| CDCA (µM) | 4.2 ± 0.8 | 0.2 ± 0.2 | 9.2 ± 2.5^b^ | 1.4 ± 1.0^c^ |
| DCA (µM) | 3.0 ± 0.3 | 0.4 ± 0.3^a^ | 1.1 ± 0.2 | 2.5 ± 1.4 |
| UDCA (µM) | 0.2 ± 0.0 | 3.6 ± 2.3 | 0.5 ± 0.1 | 10.5 ± 4.3^ac^ |
| LCA (µM) | 1.1 ± 0.2 | 1.6 ± 0.9 | 1.6 ± 0.3 | 2.6 ± 0.6 |

DKO mice at 20 weeks of age were compared. Each data represents the mean and SEM.

UDCA (–), without UDCA; UDCA (+), with UDCA.

^a^p<0.05, significantly different from Male UDCA (–) by Tukey-Kramer test.

^b^p<0.05, significantly different from Male UDCA (+) by Tukey-Kramer test.

^c^p<0.05, significantly different from Female UDCA (–) by Tukey-Kramer test.
