# Supplementary material for: Sex-, age-, and organ-dependent improvement of bile acid hydrophobicity by ursodeoxycholic acid treatment: A study using a mouse model with human-like bile acid composition
Source: PLoS One. 2022 Jul 12;17(7):e0271308. doi: 10.1371/journal.pone.0271308 (PMC9275687; doi:10.1371/journal.pone.0271308)
Supplement: Attachment — Submitted filename: Response to Reviewers.docx [file pone.0271308.s020.docx]

Replies to the comments from PLOS ONE reviewers:

Reviewer #1:

This study is very significant and comprehensive, as the new DKO model provides a new animal model with human bile acid profile to study the role of bile acids in the development of human liver diseases. Furthermore, the development of this model brings many questions that remain unanswered, which are very important to the bile acid field. There is one area in which the authors may consider is the degree of cholestasis in the female DKO mice over time, this can be done by determine the expression of several genes that are known to be altered including Mrp3 Mrp4, as well as Ostb in the liver (although the group determined CAR activity, but only narrowly). The group did determine Cyp7a1 and Cyp8b1 gene expression that were suppressed in the female mice under UDCA treatment. This is because it is possible that in female DKO mice, treatment of UDCA may exacerbate liver injury if degree of cholestasis has been more severe. Furthermore, the role of intestine FXR pathway could be determined, as UDCA is shown a FXR antagonist in the gut, which may reduce Fgf15 signaling. However, during severe cholestasis, inflammation may be more dominate than FXR pathways in the regulation of bile acid synthesis.

RESPONSE: Thank you for the reviewer’s comments. We think hepatic BA concentration is the best marker to assess a degree of cholestasis. Serum ALP activity is a useful surrogate marker for cholestasis in humans, but it is not very sensitive in mice because main mouse serum ALP isoenzyme is bone-derived one (Hatayama et al. J. Toxicol. Sci. 36:211-219, 2011). As suggested by the reviewer, we have measured hepatic mRNA expression levels of Mrp3, Mrp4, and Ostb and added them to S6 Fig. UDCA treatment tended to upregulate Mrp3 and Mrp4 expressions (Fxr independent) in both sexes, but the differences were not statistically significant. The expression levels of Ostb (FXR dependent) in females tended to be higher than those in males, but UDCA did not change the expressions significantly in both sexes. Thus, although hepatic BA concentrations, especially TLCA, were markedly elevated in female UDCA-treated DKO mice, significant upregulations of these transporters were not observed. The results suggest that TLCA may not be an effective activator of these transporters, or inflammation may be more dominant than FXR pathways in the regulation of BA transport as well as BA synthesis. We have added some discussion on the basolateral BA efflux transporters (lines 430-434).

Reviewer #2:

1. More details describing the figures and tables should be included in the legends. For example, Figure 1 has thick and thin arrows but the legend does not describe what these mean. In Table 1 there are (+) and (-), but there is no indication as to what this could mean, WT and KO?

RESPONSE: Thank you for the reviewer’s suggestions. We have revised legends of figures and tables to describe more detailed information. In Figure 1, thick and thin arrows mean the enterohepatic circulation of LCA is usually less (thin arrows) than those of other BAs (thick arrows). In Table 1, (+) and (-) mean pregnancy and no pregnancy. In Figures 2-6, (+) and (-) mean with and without UDCA treatment.

2. Isolation of microsomes should be more detailed, this method is unusual/uncommon and would be beneficial for readers to understand how it was achieved, briefly.

RESPONSE: We have added more detailed microsome preparation method to Materials and methods section (lines 155-161).

3. Tukey-kramer and Fisher are post hoc tests, meaning that an initial test (multiple groups = ANOVA) would have to be performed. The authors should review the statistical analysis methods and ensure clear indication of analysis performed in this manuscript.

RESPONSE: We agree to the reviewer’s comment, but our Fisher is not Fisher’s least significant difference test but Fisher’s exact probability test. We used the Tukey–Kramer test after one-way ANOVA but did not mention it in the text. We have revised the Statistics section in Materials and Methods (lines 178-181).

4. Histological damage should be clearly marked with arrows, stars, arrowheads, etc. Further, ductular reaction and immune cell infiltration should be stained for and quantified with IHC for CK-19 (bile duct mass), F4/80 or CD68 (macrophage marker), and CD4/8 for T cells, and MPO for monocytes. These efforts will increase understanding and impact to the readers of the results determined by the authors.

RESPONSE: Thank you for the reviewer’s valuable comments. We have marked histological damages with arrows in Figure 2B. We have confirmed the proliferation of bile ductular structure by IHC stain of CK19 (S3 Fig), infiltration of macrophages by IHC stain of F4/80 and mRNA expression levels of *Cd68* and *Cd163* (S4 Fig), and that of neutrophils and T cells by IHC stain of MPO and mRNA expression levels of *Mpo*, *Cd4,* and *Cd8* (S5 Fig). We have added this information to the Result section (lines 214-217).

5. The increased fecal excretion and pool of TBA indicate that UDCA administration in Female DKO mice may increase BA production. The authors should focus on investigating the role of CYP genes responsible for BA synthesis and the role of ASBT in the intestine of these mice (since small intestine was collected). These studies along with estrogen signaling may allow the authors to increase the impact and novel approach to this study.

RESPONSE: UDCA administration markedly increased the proportion of UDCA with a considerable reduction of CA and CDCA in the BA pool of female DKO mice. Since UDCA is not a central primary BA in mice, the increase in the BA pool in UDCA treated female DKO mice is likely due to UDCA ingestion itself, not increased BA production. Female DKO mice had larger BA pools than male DKO mice regardless of UDCA administration. We compared CYP7A1 activity and Cyp27a1 expression between the sexes (Fig.6D), but there was no significant increase in either enzyme in females. We collected the small intestines in this study but used them all to measure the BA pool. We previously measured ileal Asbt expressions in DKO mice (Ref. #8), and we found no significant difference between the sexes. In addition, unlike mice, males have larger BA pools than females in humans. Thus, the mechanism of the sex difference in the BA pool size remains unknown and is not simply an effect of estrogen.

6. It is alarming that UDCA treatment increased inflammation (seen in PCR) in the liver of female DKO. Does this indicate that the benefits of UDCA (lower miscarriage) comes at the cost of mother hepatic injury?

RESPONSE: Thank you for the reviewer’s question. Mothers do not have a hepatic injury since matings are made at nine weeks of age in our usual experiments. More prolonged UDCA treatment (20 weeks) causes liver injury in female DKO mice. If UDCA is administered only during pregnancy, older mice may be able to give birth normally without liver damage. However, we have never done that.

7. The discussion needs to be shortened to succinctly indicate where this study stands in the current literature. While it is important to emphasize key findings of this study, continuous confirmation of the previous DKO study dampens these data impact. It is recommended that the authors increase citation of works on BAs in pregnant women to identify any connections between this work and that of pregnant cholestasis.

RESPONSE: Thank you for the reviewer’s comments. We have deleted the discussion part that continuously confirms the previous *Cyp2c70* KO study (between line 402 and line 403). As suggested by the reviewer, the connection between pregnant DKO mice and intrahepatic cholestasis of pregnancy (ICP) may be interesting. Increased serum hydrophobic BAs (due to altered BA composition or cholestasis) and frequent premature delivery are common features (Ref. #19). However, UDCA treatment prevented premature delivery in DKO mice while it was ineffective in reducing adverse perinatal outcomes in patients with ICP (Ref. #22). Therefore, it is unclear whether pregnant DKO mice would be a good model for ICP. We have added these considerations and references to the Discussion (lines 374-377 and 384-386).
