# Supplementary material for: Sex-, age-, and organ-dependent improvement of bile acid hydrophobicity by ursodeoxycholic acid treatment: A study using a mouse model with human-like bile acid composition
Source: PLoS One. 2022 Jul 12;17(7):e0271308. doi: 10.1371/journal.pone.0271308 (PMC9275687; doi:10.1371/journal.pone.0271308)
Supplement: Attachment — Submitted filename: Response to Reviewers_R2.docx [file pone.0271308.s021.docx]

Replies to the comment from Reviewer #2:

The authors have addressed reviewer comments, but the CK-19 staining does not stain bile ducts. The arrows seem to indicate focal macrophage clusters instead of bile ducts. It is apparent in all images since CK-19 does not demark the bile ducts near portal veins. I would suggest that the authors review this manuscript here (PMID: 32961356) and obtain more specific CK-19 antibodies that will allow them to stain and quantify the CK-19 staining to measure bile duct mass/ductular reaction. CK-19 positivity should not exist in the sinusoidal space or within the parenchyma as it is only expressed by cholangiocytes in the liver.

RESPONSE: Thank you very much for the reviewer’s valuable comments and suggestions. We performed CK-19 staining using different mouse IgG blocking reagents and obtained better results. We have replaced the previous pictures with better ones (S3 Fig) and revised the Histopathological examination section in the manuscript (lines 133-137). In addition to CK-19, we again stained F4/80 (S4 Fig) and MPO (S5 Fig) with better conditions and replaced the previous pictures with new ones.
